# Adaptive Spatial Scheduling for Event Traffic in LoRaWAN Networks

**DOI:** 10.3390/s24072222

**Published:** 2024-03-30

**Authors:** Vassilis Asteriou, Konstantinos Kantelis, Georgia A. Beletsioti, Anastasios Valkanis, Petros Nicopolitidis, Georgios Papadimitriou

**Affiliations:** Department of Informatics, Aristotle University of Thessaloniki, 54124 Thessaloniki, Greece; vasteriou@csd.auth.gr (V.A.); kos@csd.auth.gr (K.K.); gmpelets@csd.auth.gr (G.A.B.); valkanae@csd.auth.gr (A.V.); gp@csd.auth.gr (G.P.)

**Keywords:** LoRaWAN, event traffic, adaptive spatial scheduling, TDMA, learning automata, network simulation

## Abstract

Low-Power Wide-Area Networks constitute a leading, emerging Internet-of-Things technology, with important applications in environmental and industrial monitoring and disaster prevention and management. In such sensor networks, external detectable events can trigger synchronized alarm report transmissions. In LoRaWANs, and more generally in networks with a random access-based medium access algorithm, this can lead to a cascade of frame collisions, temporarily resulting in degraded performance and diminished system operational capacity, despite LoRaWANs’ physical layer interference and collision reduction techniques. In this paper, a novel scheduling algorithm is proposed that can increase system reliability in the case of such events. The new adaptive spatial scheduling algorithm is based on learning automata, as well as previous developments in scheduling over LoRaWANs, and it leverages network feedback information and traffic spatial correlation to increase network performance while maintaining high reliability. The proposed algorithm is investigated via an extensive simulation under a variety of network conditions and compared with a previously proposed scheduler for event-triggered traffic. The results show a decrease of up to 30% in average frame delay compared to the previous approach and an order of magnitude lower delay compared to the baseline algorithm. These findings highlight the importance of using spatial information in adaptive schemes for improving network performance, especially in location-sensitive applications.

## 1. Introduction

Low-Power Wide-Area Networks (LPWAN) are an important technology in the context of Machine-to-Machine, or Machine Type Communication (M2M/MTC) and the Internet of Things (IoT), as they offer a low-cost, scalable and energy-efficient long-range service, complementary to that of earlier M2M wireless systems, which has accelerated M2M systems adoption and broadened the range of feasible applications, such as smart houses, smart grids, smart agriculture, smart cities as well as health and industrial applications [1].

One aspect of the distinct nature of M2M communications as compared to earlier telecommunications paradigms is the different traffic patterns that emerge and need to be addressed to ensure effective and scalable network solutions. Already in 2011, event-triggered synchronized traffic was considered an important component in MTC communication, and related traffic modeling followed [2,3]. However, for LPWAN networks whose basic upstream channel access mode is random access, such as LoRaWAN, event-triggered traffic can pose a significant performance challenge, due to the congestion of a higher-than-capacity volume of traffic carrying event report information in a short amount of time, leading to frame collisions in the network gateway [4].

Ensuring adequate packet delivery performance under transient higher-than-capacity traffic caused by events is key in the operation of networks where such behavior can be expected. Large-scale monitoring applications with support for alarming, such as environmental monitoring for disaster prevention and management, or smart grid management and metering, are particularly likely to suffer from the impact of event-triggered traffic on network performance, leading to degradation in the functions of the broader system’s operation due to missing valuable information.

The majority of previous research proposals for protocol improvements in LoRaWAN networks focus exclusively on improving network capacity for periodic sensor data-carrying traffic, to account for scalability issues that arise from the design of the network. For event-triggered traffic, two main types of approaches can be found in previously proposed solutions: either backoff-based (e.g., [5]) or TDMA-based (e.g., [6]). While the existing backoff-based approach is decentralized and focuses on managing the collision rate–latency tradeoff, it does not take into account the spatial aspect of traffic correlation.

On the other hand, the TDMA-based approach is a hybrid approach where, under normal traffic conditions, the network operates with basic LoRaWAN, and, only as a response to an event, the network protocol transits to TDMA in order to increase network capacity and throughput performance. This ensures that time-critical information is reliably transferred from the sensors to the application server in a way that is timely, within the limitations imposed by LoRaWAN’s data rates, and compatible with the duty cycle restrictions of ISM band regulations, as well as the LoRaWAN standard. As the occurrence of events requiring such a response is assumed to be a rare phenomenon, this approach incurs minimal energy consumption overhead on the network end devices. However, the previously proposed TDMA-based approaches adopt simplistic scheduling strategies that leave room for improvement as far as leveraging the spatial traffic correlation is concerned.

In light of these shortcomings, in this work, an effort is made to improve the performance of random access/TDMA hybrid protocols for IoT event response applications by proposing a more performant scheduling algorithm. The new algorithm is based on a learning automaton that uses network feedback information to optimize channel time allocation based on transmission origin locations and node distances. The main contributions of this paper are as follows:

1. A new scheduling algorithm for the random access/TDMA hybrid MAC protocol extension for responding to event traffic in LoRaWAN networks is proposed. The new algorithm, like Closest Next, is also an adaptive spatial scheduling algorithm. It uses a learning automaton to estimate the probability that an end device is ready to transmit an event report frame and accordingly allocates channel time to end devices in groups.
2. The paper introduces an extension to the spatially and temporally correlated event traffic model of [4,7], where spatially anisotropic events can be generated.
3. Through simulation, the new algorithm is compared to Closest Next under various traffic conditions and the relation of scheduling algorithm performance with the underlying event is explored. The new algorithm is shown to have better performance under anisotropically shaped events and equal performance under isotropically shaped events.

The rest of this article is structured as follows. In Section 2, previous solutions are discussed in more detail. In Section 3, background information about the LoRaWAN network, the event-triggered traffic model, the TDMA-based event response network extension, and adaptive spatial scheduling is given. In Section 4 the rationale and steps of the newly proposed algorithm are laid out. In Section 5 the simulation study setup is elaborated and the results are discussed. Section 6 features remarks on limitations and directions for future work. Finally, Section 7 concludes the paper.

## 2. Related Works

Regarding LoRaWAN networks, several performance-enhancing scheduling-based solutions have been proposed in the literature. A medium access algorithm for the bulk transmission of buffered sensor data is proposed in [8,9], in which the devices synchronize with the network server and receive their schedule in a two-stage handshake; later, they participate in schedule-based data collection rounds, where the schedule is determined using greedy online or offline scheduling algorithms executed centrally on the network server. Another proposal is the one proposed and evaluated in [10,11], where the synchronization procedure starts with a sync request by the end device, to which the network server responds with precise timing and smartly encoded scheduling information.

At around the same time, the S-MAC, an adaptive MAC layer and scheduler for LPWANs, was developed and published [12]. Here, the goal is to effect timing offset corrections and smart channel choice at the end device to avoid collisions and increase overall system performance. Channel configuration information is communicated to the ED as information piggybacked on the join–responseframe. The channel configuration information contains channel and transmission offset recommendations for the next transmission of the ED. Scheduling is carried out with a greedy optimization algorithm with collision minimization as its objective, and the goal is to increase the capacity and scalability for dense and large-scale sensor networks with periodic data traffic patterns.

An example of a slotted limited contention access method is Collision Avoidance Resource Allocation (CARA), as proposed in [13]. In this scheme, time is divided into slots, and each slot is composed of distinct resource blocks determined by channel frequency and spreading factor. A resource block is assigned to each device non-exclusively for each time slot in a cyclical way. When an end device needs to access the channel, it can only do so in the resource block (i.e., frequency and spreading factor) that has been assigned to it. By reducing the randomness of the channel access mechanism, the protocol achieves significant collision reduction, thus increasing capacity under conditions of regular periodic traffic. However, this MAC requires permanent global synchronization, is not collision-free, and schedules devices in a way that does not take into account the individual periodicities of data collection. Most importantly for the present work, CARA does not offer a solution for spatially correlated event-triggered traffic, as its schedule does not take into account any network feedback.

A more recent development is that of [14]. In the latter work, the signaling mechanism of the proposed scheme is similar to S-MAC, except it also explicitly communicates clock drift compensation, apart from frequency channel and transmission time offset. Frequency and time slot allocations are computed to avoid collisions and minimize the expected delay between data collection and transmission, i.e., age of information. To address the downlink scalability issue, which creates a bottleneck in the capacity of the end device signaling channel, a correction is carried out either when the frame loss or clock drift is detected to be too high. An important drawback of this approach is that enhancing the performance includes stochastic transmission suppression, which increases with data collection frequency as well as with transmission duration, i.e., spreading factor, disproportionately affecting end devices at further distances from the network server.

Another proposal is that in [15], where a Class B-based approach is used. This protocol for scheduled access uses an uplink beacon period, in addition to the downlink beacon period of Class B, as the uplink time division frame. Nodes are assigned first come, first served to groups of four whose members use different spreading factors, depending on their distance from the gateway. Based on the assigned group index, a unique slot is assigned to each device. However, this approach requires that all devices participate in the Class B scheme, which is more energy-consuming.

Overall, the above algorithms and schemes use generic heuristics to optimize for various factors, such as throughput and capacity, probability of collision, latency, and energy efficiency. While their objectives are similar, they show considerable diversity in the mechanisms employed. However, the spatial correlation of traffic, which can present an opportunity for performance optimization in the network, is not taken into consideration.

To address the potential impact of spatially and temporally correlated event-driven traffic, a few solutions have been previously proposed. In [5] the authors propose a scheme based on two ideas: (a) probabilistic suppression of transmission of event reports according to the proportion of unacknowledged transmissions, and (b) delaying event report frame transmission by an amount adaptively determined using Q-learning. The proposed scheme achieves a highly performant use of a general learning algorithm, is independent of the particular event traffic model, uses a distributed algorithm, which requires no centralized coordination, no signaling, and no extra hardware or requirements, and performs better than the baseline of random selection of event report transmission delay. However the proposed scheme requires acknowledged transmissions for the delivery of network feedback to nodes, the learning algorithm converges slowly, and with dependence on event frequency, requiring hundreds of epochs to approach maximum performance, assumes that each next event report has diminishing value and therefore deems appropriate to suppress event report frame transmission, and the use of a general learning method is shown to not scale as well with larger action sets.

In [16], the authors formulate an analytic Markov-based model, parameterized by the traffic load offered, of LoRaWAN network delay and scalability performance for a variable choice of backoff window size. After presenting the backoff window selection as a convex optimization problem, Subsequently, they propose a novel TDMA algorithm based on the optimal backoff window selection, which is shown to be a convex optimization problem. In this work, the use of the proposed scheme is evaluated under emergency traffic, which is simply modeled with a higher frame arrival rate. Thus the spatiotemporal correlation of the model we use is not taken into account either in the problem formulation or in the solution. In addition, the computation of the optimal backoff windows depends on knowing the packet arrival rates of all nodes.

In [6], a protocol for adaptive event detection and event-traffic handling is proposed, where a learning automaton executing on the network server, using network feedback information to gauge the presence of an event from frame metadata and the traffic intensity, switches between slotted random access and TDMA to improve network performance in response to traffic conditions. Apart from the performance improvement, one advantage of this approach is that event detection is built into the algorithm. However, in this work, a slotted protocol format is assumed which requires always-on network-level synchronization imposing energy and protocol overhead. In addition, the method proposed does not take into account the spatial correlation characteristics of event traffic, which may lead to lost opportunities for optimization in the TDMA phase.

In [7,17], the authors propose that traffic caused by events could be handled through a hybrid random access/TDMA MAC protocol, where the TDMA is activated only in response to an event, in order to allocate appropriate limited-contention resources for the delivery of event-report frames. The advantage of this approach is that under normal traffic no overhead is imposed on the network, or only minimal when using wake-up receivers, and the mechanism is only activated for end devices that detect the event. This approach thus trades network latency for reliability, which may be appropriate in some applications that can benefit from an alert delivery network function.

More specifically, in [7], three implementation mechanisms for TDMA over LoRaWAN are mentioned, a two-hop wake-up receiver mechanism, based on [18], a single-hop wake-up receiver mechanism, based on [19], and a novel mechanism taking advantage of the approximate implicit node synchronization caused by the event. Assuming that all event reports are important, using a TDMA-based method achieves a higher frame delivery ratio than using plain LoRaWAN, and for this reason, it is more energy efficient per delivered bit. In [17], the authors propose Closest Next (CN) scheduling, an adaptive spatial scheduling algorithm that takes advantage of the spatial correlation of the event traffic to improve network performance, however, the algorithm assumes that the event traffic is caused by a single underlying event, which affects the area around the epicenter isotropically. Due to this assumption, there are cases where the algorithm does not perform optimally.

## 3. Background

### 3.1. LoRaWAN

LoRaWAN is a network specification, first published in 2015, that was developed and promoted by the LoRa Alliance as an open Low-Power Wide-Area Networking (LPWAN) standard for the Internet of Things market. As an LPWAN, LoRaWAN stands in the long-range, low data rate and high energy efficiency part of the wireless communication systems domain, as compared to other technologies such as 802.11, Bluetooth, and cellular communications (GPRS, LTE, etc.). LoRaWAN operates in the sub-GHz unlicensed ISM bands and is characterized by robust interference management stemming from its physical layer [1]. The LoRaWAN network specification is based on a proprietary Chirp Spread Spectrum modulation technology, named LoRa, which was developed by Semtech after their acquisition of Cycleo in 2012. The purpose of the LoRaWAN specification is to enhance the industry-wide interoperability of LoRa-based IoT solutions [20].

The network architecture comprises a star-of-stars topology, with a network server in the center, directly controlling one or more gateways. In the range of those gateways, sensor nodes called the end devices are located, whose role is to monitor the surrounding environment using specialized sensor hardware and transmit reports containing some of the gathered information over the LoRaWAN network to the application server [21].

The network physical layer, LoRa, is a proprietary implementation of the Frequency Shift Chirp Modulation (FSCM) owned by Semtech. FSCM is a spread spectrum technique where symbols take the form of frequency sweeps, i.e., chirp signals, modulo the channel bandwidth, and each symbol value is encoded as the frequency displacement of the chirp relative to the reference chirp signal [22]. The transmission parameter that plays the most important role in LoRa transmission performance is the spreading factor, which controls the transmission chirp rate. Increasing the spreading factor by one increases the transmission range but doubles the transmission duration, thus halving the data rate. Notably, transmissions on different spreading factors are mathematically orthogonal and, practically, this results in the formation of independent logical channels for each spreading factor in the same frequency band [23]. The design of the physical layer explicitly trades the data rate for increased receiver sensitivity and thus can achieve ranges of a few kilometers in urban areas and about 10–15 km in rural areas, albeit only at data rates up to 5 kb/s [1].

At the MAC level, uplink communications follow a duty-cycled random access protocol. For downlink communications, the LoRaWAN standard defines three service classes, implemented as different protocols [21]. In Class A, downlink transmissions can only occur in predefined reception windows shortly after an uplink transmission. In this way, the end devices can spend their entire time in sleep mode, except when they are ready to transmit data reports, thus conserving energy and for battery-powered devices, longer battery lives are achieved. Frames may be unconfirmed, which may be transmitted more than once but require no acknowledgment, or confirmed, which are transmitted until an acknowledgment is received in the reception windows.

In Class B, except for the Class A reception windows, additional downlink reception windows, named ping slots, are defined. The ping slots are temporally specified relative to periodic beacon frames, transmitted synchronously by the gateways, whose function is to transfer control information as well as facilitate synchronization between the gateway and the end devices. In Class C, the end devices are always listening. Thus, the three service classes offer different options in the downlink latency versus end device energy consumption trade-off.

### 3.2. Event-Triggered Traffic Modelling

An IoT monitoring system in normal conditions will use its sensors to periodically collect measurements of its environment and transmit reports to the system’s central server for processing and analysis. However, another possibly desirable function of an IoT monitoring sensor network is to forward event reports, which may be important for alerting functionality. When event-related network activity occurs due to external events that are observable by a large number of devices, a high volume of spatially and temporally correlated traffic is generated.

This section describes the event-triggered traffic model used in this work. First, a short exposition of the model’s evolution is given, focusing on how it has been used in the research literature to characterize the performance of random access-based IoT networks, and more specifically LoRaWAN, under event-triggered traffic. Subsequently, the definition of the model employed in this work is laid out in detail, covering the temporal and spatial propagation modeling as well as the rationale of the model design.

An initial event-triggered traffic modeling attempt is based on the Beta distribution model specified in [24]. In order to convert the aggregate traffic model to a source model, the authors of [2] used a Coupled Markov Modulated Poisson Process (CMMPP) formulation, whereby the node traffic intensity is the state of a Markov chain with time-varying transition probabilities, which in turn is not independent of that of other nodes but instead is determined by an underlying background process, which couples the Markov chains and also models the spatial and temporal correlation of the traffic. The correspondence of the CMMPP model with the Beta distribution aggregate model is further confirmed in [25]. Subsequently, in [4] a LoRaWAN network under the CMMPP model is simulated, which shows severe performance degradation under event-triggered traffic.

In the CMMPP model definitions used in [4,25], the node remains in normal traffic state at all times except for a particular moment, which is determined from the event propagation delay, when a node transits to the event traffic state. In the event state, the node generates a single frame with probability 1 and subsequently immediately returns to the normal traffic state. The above interpretation of the CMMPP model led the authors of [17] to use discrete event simulation-based traffic generation and bypass the need for explicit simulation of the CMMPP.

The model used in this work is a source-based traffic model that samples event-triggered traffic arrival times and nodes in such a way that the arrival events are spatially and temporally correlated. The distinct characteristic of this model, compared to previous approaches, is that the areas affected by the event, i.e., the spatial regions in which the traffic is generated, are not necessarily isotropic but can have random non-isotropic shapes. Furthermore, both the event size and its deviation from the isotropic shape are controllable. These distinct characteristics are important for the demonstration of this work’s novelty and the proposed algorithm’s performance.

In the model employed in this work, an event is defined by

1. its origin $(t_{e},$$p_{e})$, where $t_{e}$ is the time of occurrence and $p_{e}$ is the point in space where the event originates,
2. its effect area, defined via a function Ψ(x), which is equal to the probability that an end device located in point x will detect and respond to the event, and
3. its propagation velocity *v*, which determines the time delay $\Delta t=∥x-p_{e}∥/v$ between the initial occurrence of the event $t_{e}$ and its detection by an end device positioned at point x.

In the literature, the area of effect function Ψ is found in forms that depend only on the distance $d=∥x-p_{e}∥$ between the end device and the event origin, and it is termed the spatial correlation function [4,25]. The above form, therefore, assumes that the event affects a region around the epicenter isotropically, i.e., the effect is rotationally invariant statistically. Consequently, it does not take into account events with irregular areas of effect, which may be affected by physical and geographical features of the underlying monitored field.

It should be noted here that events do not always follow a typical break-out pattern. Instead, there exist cases, like spreading fires, that are noisy and potentially chaotic systems in which transitions in dynamics are difficult to predict and demand a novel way of consideration [26]. Following the same rationale, in [27], a heterogeneous data fusion algorithm for detecting a fire in a defined area was proposed, which enables ignoring unnecessary and incorrect data that may influence the reliability of the detection. The magnitude of the event propagation is estimated at a second level using a Fuzzy Inference System. Nontrivial event occurrence and propagation were also under investigation in the work of [28], where as time passes, hot spots appear and expand, merge, move, contract, and finally disappear in the field creating some non-symmetrical event propagation patterns. Motivated by the above approaches, in this work, a more general approach is taken, in which the model caters also to cases where the event affects an arbitrary spatial section of the monitored field or the case of multiple simultaneous events.

In order to define a model that delimits arbitrary spatial sections, an overall area of effect of an event is defined in terms of smaller components [7]. Let $\Psi_{1},\Psi_{2},\cdots$ be component area-of-effect functions, i.e., $\Psi_{i}\left(x\right)$ is the probability that an end device positioned at point x will be affected by the *i*-th component of the event. Then, the probability that the same end device will be affected by at least one of these components is

(1) $$\Psi \left(x\right)=1-\prod_{i}\left[1-\Psi_{i}\left(x\right)\right]$$

The generality of the above construction is evident considering that combining, for example, radial step functions centered at different locations, arbitrary and general event effect area shapes inside the monitored field can be modeled. Consequently, in the above formulation, as the event can propagate away from the origin by taking arbitrary shapes in space, the more general term “area of effect” is preferred and will be used in the remainder of this paper.

This leads to the following strategy for sampling a randomly shaped area of effect: first, a set of points can be sampled such that each point is placed at a distance of at most *z* from at least one other point in the set, and second, the components of the area of effect function Ψ can be radial step functions centered about the points of the above set with a radius such that a contiguous area is formed:

(2) $$\Psi_{i}\left(x\right)=\left\{\begin{array}{cc} 1 & \mathrm{if}\;\;||x-x_{i}\left|\right|\leq z\\ 0 & \mathrm{otherwise} \end{array}\right.$$

To generate the random point set, an algorithm based on the Poisson disk sampling technique is used. Poisson disk sampling is a type of blue-noise point set generation algorithm. Point sets of this kind are characterized by a regular geometric pattern (as opposed to a clustered one), as well as a uniform minimum distance from any point to its closest neighbors. The term “blue noise” arises from the fact that the spectrum of such point sets resembles a high-pass filter.

Specifically, we used a modified version of Bridson’s Fast Poisson Disk Sampling algorithm [29]. In the original algorithm, two sets are used, the generated points set and the active list. Both are initialized with the same randomly generated initial point. While the active list is not empty, a point *x* is chosen from the active list and up to *k* attempts to generate a new point are made. If an attempt is successful, the new point is added to both the point set and the active list, otherwise, the point *x* is removed from the active list. Given the point *x* from the active list, a point generation attempt consists of uniformly sampling a point from the annulus of radius between *r* and 2r around *x*, but it is rejected if it is outside the field, or if it is closer than *r* to another point in the generated point set. By setting z=2r in the component functions a contiguous area of effect is obtained.

The modifications made are the following:

1. The algorithm stops early, as the goal is not to generate a random field that fills the entire space, but rather just has an anisotropic boundary. This is implemented by only sampling up to *N* points, where *N* is drawn from a Poisson distribution with a mean equal to the event size factor *s*.
2. A directional bias of strength *d* in the sampling of each next point from the active set is used. In particular, given the direction $\hat{u}$, instead of picking an active set point with equal probabilities, the probability of each active set point to be chosen is $\pi_{i}=y_{i}/\sum_{j}y_{j}$ whereby
  (3) $$y_{j}=\exp\left(d\frac{p_{j}-\min_{k}p_{k}}{\max_{k}p_{k}-\min_{k}p_{k}}\right),\;\;\;p_{k}=\hat{u}\;\cdot \;x_{k}$$
  With this formulation, points that lie more towards the direction $\hat{u}$ are favored more as *d* increases.

### 3.3. TDMA MACs for Event Response in LoRaWAN Networks

The above event model, but also event models for IoT and MTC more generally, are based on the assumption of spatially and temporally correlated traffic. Given that in LoRaWAN networks uplink frame transmissions are subject to a random access MAC protocol, implementing an event response protocol that is spatially targeted with managed delay can be challenging. In this work, a TDMA mechanism over an underlying two-hop wake-up receiver architecture, as described in [7], is employed. The core idea is that independent of the event detection mechanism, the network can start TDMA frames after detecting an event so that event report frames have a channel with higher capacity, which however is also more energy-consuming, through which they can timely and reliably reach the application. In the above work, several alternative mechanisms for such an implementation were explored and evaluated.

In this work, the On-Demand TDMA (ODT) network extension, first proposed in [18], was chosen. In this extension, the LoRaWAN end devices are additionally equipped with wake-up receivers, which enable the end devices’ deep sleep to be interrupted. Due to the short range of the wake-up receivers used in the prototype, which are required for the system to remain energy efficient and not drain the end device batteries, the network is segmented into clusters of neighboring nodes, such that all end devices in a cluster are in the range of the wake-up transmitter of a cluster head node. In response to a wake-up beacon transmitted by a cluster head, the end devices may transmit a message in a predefined time slot determined during network initialization. This mechanism thus inverts the communication modality from push-based to pull-based, where the initiative of communication is transferred from the end devices to the network. Finally, the cluster heads are considered to be wall-powered and connect to the network server via the LoRaWAN network as Class C devices in order to reduce the On-Demand TDMA mechanism turnaround time.

When considering the above scheme as the mechanism for the adaptive consecutive scheduling of multiple clusters to be used in the event–response scenario, the issue of duty cycle limitations in LoRaWAN networks arises, which is relevant for both downlink and uplink transmissions. Assuming the European Union as the regulatory domain, the specification of the competent authority for radio frequency regulation, ENISA, includes various duty cycling limitations to various sub-channels in the 868 MHz ISM band [30]. These specifications limit the total time on air of any transmission during any 1 h long observation window. An assumed default downlink channel at 869.525 MHz, where a 10% duty cycle applies, is used to signal the cluster heads; the gateways have 360 s/h to be used at their discretion, which is more than enough time given the spacing between subsequent downlink transmissions [7].

For uplink communications in the scheme proposed in this work, each end device is required to perform up to a single additional transmission, which is well within ENISA limitations, given the one-hour observation period criterion and assuming devices operate reasonably below the duty cycle limit. Additionally, the LoRaWAN standard specifies two cases of duty cycle restrictions to be observed, beyond those in regional regulations. [31] The first case is the network duty cycle policy, which is an optional mechanism controlled per end device by the network operator. If an ODT-enabled LoRaWAN network has a duty cycle policy, an exception could be made for ODT transmissions, as in ODT it is the network server that, in effect, triggers the transmissions.

The second mechanism described in the LoRaWAN standard is the retransmission backoff provision, which is mandatory and applies to synchronized persistent traffic, i.e., frames that both (a) require a network server acknowledgment or answer frame, without which the uplink will be retransmitted, and (b) can be event-triggered in a way that causes large-scale network synchronization. In this work, the way ODT is used for adaptive spatial scheduling for event-triggered traffic implies that the event-triggered frames are retransmitted up to once through the ODT mechanism and no further retransmissions occur. Thus, this restriction does not apply.

### 3.4. Adaptive Spatial Scheduling

Adaptive Spatial Scheduling is any time division-based medium access protocol where network node scheduling takes into account both (a) spatial relations between nodes, and (b) network feedback information. Thus, the distinguishing feature of Adaptive Spatial Scheduling as a protocol family compared to other time division scheduling algorithms is not its objective, but the type of network information utilized towards the goal of network performance optimization. For a network of *n* nodes using an Adaptive Spatial Scheduling protocol, a metric distance matrix $D={\left(d_{ij}\right)}_{n\times n}$ is defined, where $d_{ij}$ is the distance between nodes *i* and *j*. A metric distance matrix is a symmetric non-negative matrix with diagonal elements equal to zero, and whose elements satisfy the triangle inequality. This matrix encodes the spatial relation between the nodes of the network and is independent of the network topology graph. In this work, the distance matrix D is assumed to be constant and known by the network protocol. In the most general case, the matrix D may be non-Euclidean; for example, the nodes may be wireless nodes that monitor the electric grid, where the underlying spatial structure encoded in the distance matrix is the electric grid topology. In the forest monitoring application domain, $d_{ij}$ is naturally defined as the Euclidean distance between nodes *i* and *j*. In the Euclidean case, an equivalent representation of the spatial relations of the network nodes is the set of node locations [32,33]. Thus location-based scheduling algorithms are a subset of adaptive spatial scheduling algorithms.

Closest Next Scheduling is a *location-based* Adaptive Spatial Scheduling algorithm for the event response application that is described above. Closest Next works by estimating the event epicenter and scheduling the candidate that is located closest to the epicenter estimate. The epicenter estimate is defined as the centroid of the positions of all the end devices that have recently reported the event. In this way, the algorithm takes advantage of spatial relations assuming that sensors closest to the epicenter are more likely to have been affected by the event. An IoT system using Closest Next scheduling under event-triggered traffic performs better in terms of delivery ratio compared to a random access protocol, and smaller delay compared to a round-robin scheduler [17].

After the initial evaluation of the Closest Next scheduling algorithm, it became clear that its performance is optimal only under isotropic events. When the event area of effect has an irregular shape or multiple independent events take place, the algorithm is no longer optimal as it takes more time than necessary to schedule all relevant end devices, and the advantage of using spatial scheduling compared to round-robin scheduling diminishes, as end devices unrelated to the event may be scheduled. In the sections that follow, a new, *distance-based* scheduling algorithm that outperforms Closest Next in the above conditions is presented and evaluated.

## 4. Adaptive Spatial Scheduling Using Learning Automata

In this section, the Learning Automaton Exploration (LA-EXP) adaptive spatial scheduling algorithm for use in event response TDMA protocols is introduced. The algorithm uses network feedback information in combination with knowledge of the location of devices to optimize channel time allocation under spatially and temporally correlated traffic. To this aim, a learning automaton is used, which is a machine-learning technique, similar to reinforcement learning, that improves its performance during and through interactions with its environment. This is achieved by receiving feedback signals from the environment, updating the automaton’s internal state according to predefined processing rules, and producing an action that is applied in the environment. Due to the lightweight nature of this type of algorithm, it has found many applications in networking, such as [34,35]. In the following subsections, first, the reasoning of the algorithm is introduced in detail, and then a proposed implementation is described. Table 1 summarizes the notation used in this work.

### 4.1. Algorithm Description

**Figure 1:**
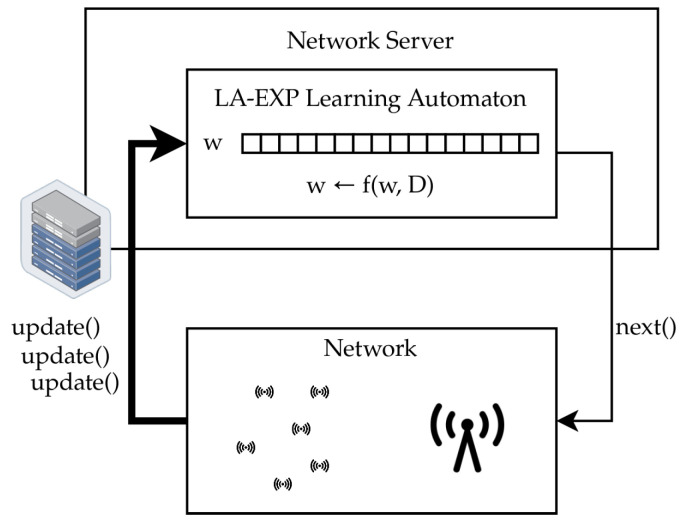
The feedback loop of the LA-EXP learning automaton. The LA-EXP computes the next action, i.e., which cluster should be scheduled next. The network server then applies this action to the network, which may result in event-related uplink transmissions. These transmissions are relayed to the LA-EXP learning automaton, and thus constitute its environmental feedback, closing the feedback loop.

**Figure 2:**
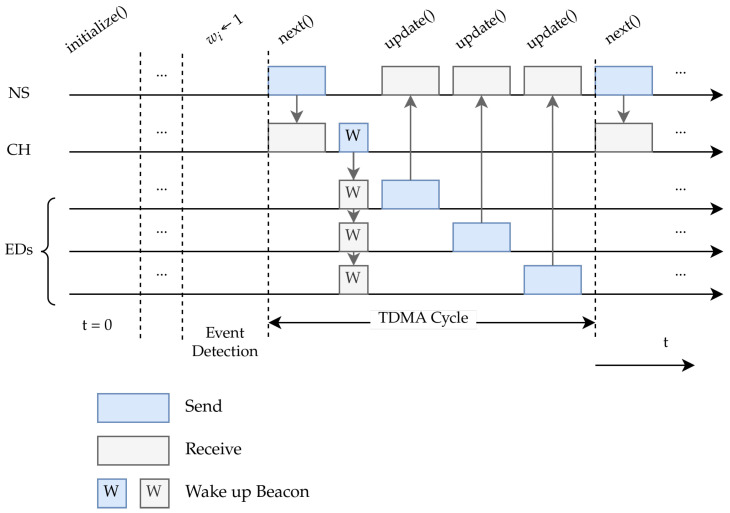
Example runtime of a LA-EXP instance. At time t=0 the network is initialized. After an arbitrary amount of time, an event is detected and the network server marks nodes estimated to be in the area of effect by setting their weight to 1. Then, the sequence of TDMA cycles starts. In a TDMA cycle.

The adaptive algorithm used is a learning automaton, which uses network feedback information to construct a model of how likely each node is to have fresh event-related information to transmit. The model output is the node cluster most likely to be ready to transmit fresh information and, based on this, the network server directs the On-Demand TDMA scheduling mechanism. In this way, the model’s result drives subsequent changes in the environment by potentially causing further event-related frame transmissions which drive further updates to the automaton state. Thus, as more event reports come in, new areas affected by the event become known to the model and, through the use of the distance matrix, the learning automaton explores the monitoring field for event-related traffic. The feedback loop between the network and the learning automaton is illustrated in Figure 1, while Figure 2 contains a schematic representation of the time evolution of the TDMA scheme at runtime.

Let *S* be the set of nodes, and N=|S| be the number of nodes. Also, let w be a vector of dimension equal to *N*. The elements of w are weights $w_{i}$ associated with each node i∈S and represent an estimate of the probability that node *i* has detected the event and is ready to transmit a novel event report. Initially, w=0 as no devices are suspected to be ready to transmit fresh event-related information. As mentioned above, the algorithm is assumed to operate on top of the ODT architecture which implies that the nodes are organized in groups, the ODT clusters, that can be scheduled as a unit. We use the term group in the context of the scheduling algorithm to distinguish scheduler groups from the ODT clusters.

Let *G* be the set of groups and $S_{g}\subseteq S$ denote the set of nodes in group g∈G. Then for each group, the estimated probability that at least one node in the group is ready to transmit fresh information is:

(4) $$q_{g}=1-\prod_{i\in S_{g}}\left(1-w_{i}\right),\;\;g\in G$$

Given the above probability estimates, the set $E=\{g\in G/q_{g}\geq q_{t}\}\subseteq G$ of groups eligible for scheduling can be defined, where $q_{t}$ is a threshold value and a parameter of the algorithm. At each scheduling step, the group in *E* with the highest weight, i.e., probability of being ready to transmit new information, is selected amongst all groups, and the network server signals the corresponding nodes to transmit their event reports. If E=⌀, the algorithm terminates.

As new event reports arrive at the network server, either before or during the TDMA phase, the weight vector is updated accordingly in order to reflect the new information. The new weight of node *i* after an event report arrives at the network server sent by node *n* can be then expressed as a convex combination of 0 (penalty), 1 (reward), and its current value $w_{i}$ (neutral update):

(5) $$w_{i}\leftarrow \left(1-u\left(t_{i}\right)\right)\left(1-\eta_{in}\right)\;\cdot \;w_{i}+\left(1-u\left(t_{i}\right)\right)\eta_{in}\;\cdot \;1+u\left(t_{i}\right)\;\cdot \;0$$

In the above expression:

1. $u\left(t_{i}\right)\in \left[0,1\right]$ represents the time penalty of node *i* and delivers a penalty to nodes that have recently been allocated the channel to transmit an event report, and thus are likely to behave as in the recent channel allocation, i.e., either send already known information or send no information at all. The time $t_{i}$ is the time since the channel was allocated to the node *i* in a TDMA cycle. For simplicity, in this work, the following time penalty is assumed:
  (6) $$u\left(t\right)=\left\{\begin{array}{cc} 1 & \mathrm{if}\;\;t<+\infty \\ 0 & \mathrm{if}\;\;t=+\infty \end{array}\right.$$
  where, by convention, t=+∞ if the channel has not been allocated to the group containing device *i*. With the above formula recently scheduled groups are excluded from the scheduling. Other options for more complicated system models are step functions or exponential decay functions. A step function, for example, could exclude only sensors to which time on the channel has been recently assigned.
2. the parameter $\eta_{in}\in \left[0,1\right]$ is the conditional probability that, during a single event, given that node *n* has generated an event report, node *i* has also generated an event report.
  (7) $$\eta_{in}=P\left(E_{i}|E_{n}\right)$$
  The guiding assumption here is that if a node has transmitted an event report, then its *m* nearest neighbors are also probably ready to transmit such a report, with a probability decreasing for larger distances from the node. Thus, *m* is a parameter of the algorithm. To encode this assumption, the η parameter is modeled as follows:
  (8) $$\eta_{in}=\varphi \left(\frac{d_{in}}{r_{n;m}}\right)$$
  where:
  - $d_{in}$ is the distance between end devices *i* and *n* as encoded in the distance matrix D of the network,
  - $\varphi :\left[0,+\infty \right)\to \left[0,1\right]$ is a non-increasing function since nodes closer to *n* are more likely to also have been affected by the same event that devices at larger distances,
  - φ(x)=0 for x>1 so that only a limited number of weights need to be updated, and
  - $r_{n;m}$ is the distance of node *n* from its *m*-th closest neighbor node, to guarantee weight updates of at least *m* weights.
  In the above formulation, it is clear that the nodes updated after the reception of an event reported from node *n* correspond to the vertices adjacent to *n* in the *m*-nearest neighbor graph induced by the distance matrix D. In this way, the algorithm remains performant in the case of the variable density of sensor placement in the field by guaranteeing that, for each update, the *m* closest nodes are rewarded regardless of density and thus considered for future channel allocation. The specific φ used is $\varphi \left(x\right)=1-0.7\;\cdot \;x^{2},\;\left|x\right|<1$.

### 4.2. Implementation Considerations

From the coding perspective, the implementation of the algorithm consists of two main routines: update(n) which is executed whenever an event report arrives at the network server from node n and updates the weight vector w, and next(), which is executed at the beginning of each TDMA cycle to determine if there is need to schedule another group and if so which one.

The prototypical implementation of this work is shown in Algorithm 1. Each update() call requires a partial sorting of the nodes in order to find the *m* nearest neighbors of the “updated node” n. This sorting is carried out on a separate buffer and requires a single pass over a column of the distance matrix. Its worst-case runtime complexity is therefore O(Nm). During a single TDMA cycle, which consists of uplink transmissions of some of the notified devices, the update() function runs once per event-related transmission. As the node density of the network increases, the total number of update() calls increases proportionally in the worst case. Thus, in order to reduce the load on the network server, very high-density deployments might opt to increase cluster head density as well. This would provide the additional benefits of better coverage and, spatially, more fine-grained group formations and thus scheduling.
**Algorithm 1** Learning Automaton Exploration Scheduling.Given the following:
Set of nodes *S*Mapping of each node to its (unique) group g:S→GGroup member sets $S_{g},\;g\in G$Distance matrix D 1**function** initialize():2 **w** ← **0**3 **u** ← **0** 4**function** update(n):5 // Initialize a sorting buffer N of size m with (Ø, ∞)6 // node index-distance pairs7 // N is indexed from 0 to *m* − 18 (*N*)_*m*_ ← [(Ø, ∞), (Ø, ∞), …, (Ø, ∞)]9 10 // sort the closest neighbors from S into the buffer11 **for each** *i* ∈ *S*:12  **let** (*x*, *d_xn_*) = *N*_0_13  **if** (*d_in_* < *d_xn_*) **then** *N*_0_ ← (*i*, *d_in_*)14  **for** *k* = 1, 2, 3, …, *m* − 1:15   **let** (*j*, *d_jn_*) = *N_k_*16   **if** (*d_in_* < *d_jn_*) **then** swap(*N*_*k*−1_, *N_k_*)17   **else break**18 19 // N contains the m closest neighbors20 // of n sorted in descending order (and node n)21 **let** (*x*, *d_xn_*) = *N*_0_22 *r*_*n*;*m*_ ← *d_xn_*23 24 // Update the weights corresponding to25 // the m closest neighbors of n26 **for each** (*i*, *d_in_*) ∈ *N*:27  *η_in_* ← *φ*($\frac{d_{\mathrm{in}}}{r_{n;m}}$)28  *w_i_* ← (1 − *u_i_*)[(1 − *η_in_*) · *w_i_* + *η_in_*] 29**function** next(): → g_*_ ∈ *G* or Ø30 **q**^*c*^ ← **1**31 **for each** *i* ∈ *S*:32  $q_{g(i)}^{c}$ ← $q_{g(i)}^{c}$ · (1 − *w_i_*)33 **q** ← **1** − **q**^*c*^34 35 **for each** *g* ∈ *G*: **if** *q_g_* < *q_t_* **then** *q_g_* ← 036 **if** (**q** == **0**) **then return** Ø37 g_*_ ← argmax_*g*_ **q**38 **for each** *i* ∈ *S*_g^*^_: *u_i_* ← 139 **return** g_*_

The next() function requires a single pass over the node-weights vector to reduce it to the group-weights vector, one pass over the group-weights vector to filter groups and find the maximum, and, if a group is selected for scheduling, a pass over the nodes of that group to update the penalty vector. Thus, this function also has a worst-case runtime complexity of O(N).

One important aspect of this paradigm, which applies to this implementation as well, is that it requires some basic feedback-based cooperation between the network and application servers. This is because the application server alone is responsible for inferring that a packet is event-related and letting the network server know accordingly, but the network server alone is responsible for controlling the underlying TDMA mechanism that drives the scheduled access-based network response. In this sense, real-world deployments could greatly benefit from allowing at least the part of the application server that delivers protocol feedback information to the network server to run as close to the latter as possible, preferably on the same machine. The goal of this would be to eliminate possible latency or availability issues in the network server–application server link due to the best-effort nature of the Internet. In short, the incorporation of edge computing concepts in the LPWAN architecture can provide tangible benefits in real-world deployments.

## 5. Simulation and Results

In order to assess the validity and performance of the newly proposed scheduling algorithm, an in-house discrete event LoRaWAN network simulator with support for event-triggered traffic, as well as a prototype implementation of the event response TDMA MACs and the Round Robin (RR), CN and LA-EXP scheduling algorithms was used, based on extension of previous work [7]. The simulation study focuses on two objectives: (a) validating that the algorithm functions as intended, and (b) showing that LA-EXP achieves improved performance compared to the earlier-proposed CN scheduling scheme.

### 5.1. System Model

In this section, the system model used in the subsequent analysis is described in detail. An urban region shaped as a circular disk of radius 2.5 km constitutes the monitoring field, where sensor nodes are deployed at various heights and depths inside buildings, with a node placement density of *λ* = 500 nodes/km^2^, unless otherwise specified in specific simulation runs. Each node is connected as an end device to a single-gateway LoRaWAN network. The network operates under the LoRaWAN EU868 regional parameter set for the physical layer, using three uplink channels. The wireless transmissions are subject to Hata path loss, as well as building penetration loss according to the scenario 1 specification in [24], following the modeling of [4,7]. The end devices connect to the network using the minimum spreading factor such that the received power from a 14 dBm transmission is above the gateway sensitivity [36,37], while devices out of range are pruned from the simulation. The same sensitivity values as in [4] were used, which are sourced from LoRa hardware manufacturer datasheet material. End devices always transmit at 14 dBm.

Since the ODT LoRaWAN network extension [18] is used to support the adaptive TDMA response, additional cluster head nodes need to be simulated. Cluster heads are placed at the vertices of a hexagonal lattice, such that adjacent cluster heads are 250 m away from each other, while nodes are assigned to the group whose cluster head node is the closest. Wake-up beacon transmissions are assumed to always be received successfully by the wake-up radios. Since different nodes are connected using different spreading factors, and given the orthogonality of transmissions of different spreading factors on the same frequency channel, each ODT scheduling period consists of simultaneous time slots coexisting across the different frequency channels and spreading factors, which are assumed to be assigned when end devices first join the network.

Each simulation run consists of a single event episode. The episode starts at time *t* = 10 s and is detected by the network server at time *t* = 15 s. The propagation velocity is *v* = 4000 m/s without loss of generality, as it does not affect the performance of the event response TDMA scheme [7]. Upon detection, the network server enables the TDMA response MAC protocol, using either RR, CN or LA-EXP as the scheduling algorithm. The round-robin scheduler, which is included as a baseline approach, uses a fixed predefined list containing all the clusters in randomized order as the schedule used in the TDMA. The study focuses exclusively on event-triggered traffic, and thus regular periodic traffic is not enabled.

The metrics under which the algorithms are evaluated are the *frame loss ratio* and the *average frame delay*. In a single simulation instance, the frame loss ratio is measured as the percentage of all generated event report frames that are not received by the network server. The delay of a single frame is the time between its generation time, which is equal to the time of event detection by the node, and the time the frame is delivered to the network server. The average frame delay is computed as the average value of frame delays of all frames that are delivered in a single simulation instance. Owing to the stochastic nature of the model, the simulations need to be run multiple times to estimate the distributions or the expected values of the above two metrics.

**Figure 3:**
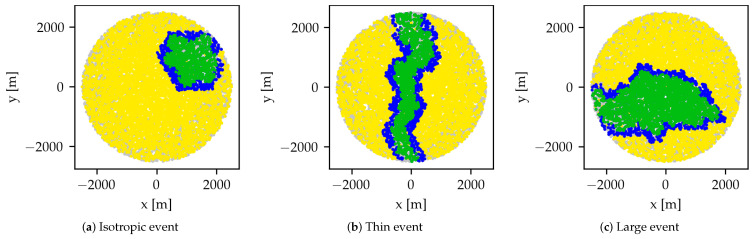
Illustrations of the event types under which the scheduling algorithms are evaluated in this paper. Each diagram shows the final simulation state with LA-EXP. Every point in each diagram is a node, gray for pruned, yellow for idle, green for affected by the event and scheduled by the algorithm, and blue for scheduled but not affected by the event.

The simulations are carried out under three sets of parameters of the random event generation algorithm, corresponding to isotropic, thin and large events respectively. The parameterization sets are shown in Table 2 while Figure 3 shows characteristic examples of events from the three categories. The isotropic and thin types represent cases where the performance of CN and LA-EXP are expected to coincide and diverge sharply respectively, while the large event type represents realistic large-scale event conditions. For the RR scheduler, since it is not adaptive, only the size of the event can have an impact on scheduling performance.

### 5.2. Results

In this section, the results of the simulation study are presented. The scheduling algorithms were subjected to varying network densities, event types, and parameter values in order to obtain simulation results regarding performance comparisons across a range of conditions.

**Figure 4:**
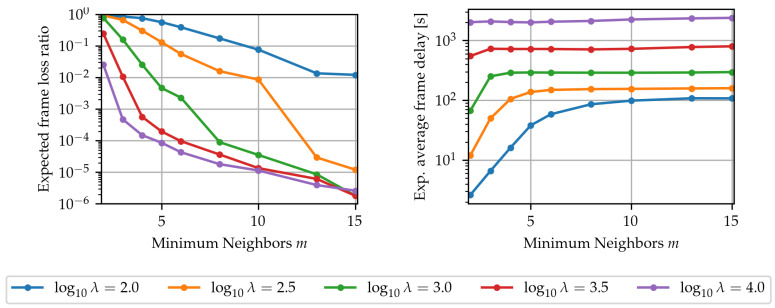
Effect of the minimum neighbors’ parameter *m* of LA-EXP on the expected values of the frame loss ratio and average frame delay under deployments of varying node density λ (in $\mathrm{nodes}/{\mathrm{km}}^{2}$).

First, to assess the impact of the minimum updated neighbors parameter *m* on the LA-EXP algorithm’s performance, the simulation was run n=500 times for values of m∈[2,15] and for node densities $\lambda =10^{2+k/2},\;k=0,1,\cdots 4$, where λ is in nodes/km^2^. The event type used was thin events. The results, shown in Figure 4, highlight that increasing the minimum updated neighbors parameter *m* generally leads to higher frame delivery performance, but the impact of the *m* parameter appears to be scale-dependent, with smaller-scale networks requiring a higher number of updated neighbors for the same level of frame loss performance. In addition, for each given level of network deployment density λ, there seems to be a cutoff point in the minimum neighbors parameter such that further increases yield little frame delivery benefit for a marginal average frame delay cost. Thus, a simple strategy for selecting the minimum neighbors parameter under uncertain conditions is to use a “generous” value, e.g., m=10, and only decrease it if there is a significant expected runtime performance benefit.

**Figure 5:**
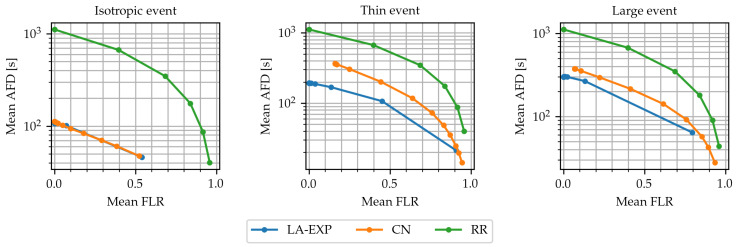
Comparison of Mean Frame Loss Ratio–Mean Average Frame Delay characteristic curves of CN and LA-EXP scheduling for the three event types.

Next, to evaluate the simulation performance of RR, CN, and LA-EXP scheduling relative to each other, the simulation was run using all algorithms for each of the three event types and varying values of the algorithms’ parameters. For the RR scheduler, the parameter varied was the total duration of the TDMA phase, i.e., the scheduling was allowed to run only up to a duration $T_{\max}$, ranging in logarithmic scale from 100 s to 5000 s before terminating. For CN, the maximum idle time parameter $I_{\max}$ was given values ranging in a logarithmic scale from 2 s to 1000 s, and for LA-EXP the minimum neighbors parameter took the values of m=1,2,3,⋯,10. For each unique parameter set, the simulation was run n=1000 times. The diagrams in Figure 5 show the expected average frame delay–expected frame loss ratio characteristic curves for the compared algorithms over the ranges of their respective parameters, as specified above, under each event type.

The diagrams highlight that for all algorithms, their parameters offer a tradeoff between average frame delay and frame loss ratio. For the adaptive spatial algorithms, a possible reason is that both CN and LA-EXP are prone to early stopping when the stopping condition is too strict. Relaxing their stopping condition by increasing the value of the respective parameter could therefore result in more frames being delivered and, thus, inevitably to a greater average frame delay. For the RR scheduler, this behavior is expected, as limiting the total TDMA time reduces the probability that all affected clusters are scheduled. To achieve maximum frame delivery performance, the RR scheduler requires that all clusters are scheduled, yielding the same average frame delay at minimum frame loss rate, regardless of event type for the given node deployment density, while the delay is about an order of magnitude larger than LA-EXP.

The adaptive algorithms appear to outperform the baseline algorithm, as seen in Figure 5, since the RR algorithm requires a greater average frame delay to achieve the same frame loss ratio. This is because the adaptive algorithms take advantage of the spatial and temporal correlation of the event-triggered traffic to optimize their schedule. As the area affected by the event becomes less symmetric, CN scheduling requires that its parameter be increased, i.e., its termination condition be relaxed, in order to maintain the same level of performance. On the other hand, LA-EXP scheduling consistently achieves a frame loss rate of the order of <10^−1^ for values of m>5, consistent with the results of Figure 4. Importantly, it is clear that, in this use case, LA-EXP scheduling is at least as performant as CN, since it can achieve the same level of frame loss ratio for a smaller or, at most, the same average frame delay.

**Figure 6:**
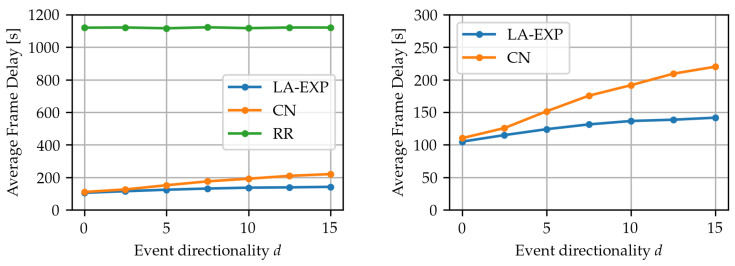
Effect of randomly generated event directional strength factor *d*, which is a proxy for the event anisotropy, on expected average frame delay. Left diagram: comparison of the three algorithms. Right diagram: detail of left diagram.

To validate and quantify the hypothesis that the two algorithms’ performance diverges when anisotropy is introduced in the underlying event, the simulation was run for events generated based on the isotropic event type, but with varying directional strength factor d∈[0,15]. The algorithm parameters that minimize the frame loss ratio in each algorithm were chosen, to render the results about delay comparable. For each algorithm and value of *d*, the simulation was run n=2000 times and the expected average frame delay was estimated from the results. The result, shown in Figure 6, highlights that as the directional strength factor *d* increases, the average delay of CN increases at a higher rate than that of LA-EXP. This further illustrates the point that CN becomes less efficient than LA-EXP as the anisotropy of the event’s area of effect increases, but the two adaptive algorithms have similar performance for isotropic events. On the other hand, the performance of RR, as expected, does not show any dependence on the shape of the underlying event.

**Figure 7:**
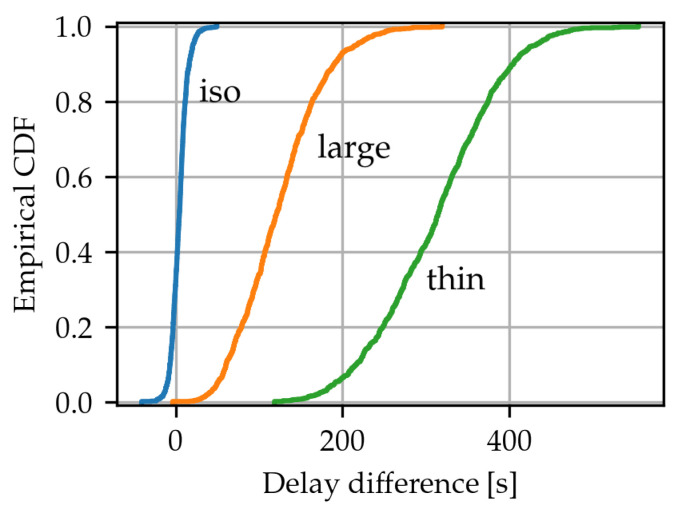
Distribution of the average delay difference between the two algorithms under the three considered event types.

While the results in Figure 5 focus on the average performance of the compared spatial scheduling algorithms over wide parameter ranges, the question of variance remains. To assess whether any variance in performance plays an important role in the overall outcome, the distribution of the per-case average delay difference of the algorithms was estimated in the cases of each of the three event types. To do this, for each of the three event types, n=1000 unique events, as determined by the simulation seed and event generation parameters, were simulated using the CN and LA-EXP algorithms. As before, for algorithm performance metrics to be comparable, the parameters of both algorithms are such that minimal packet loss occurs. Then for each unique event, the difference in the average frame delay between the schedulers was computed. Figure 7 shows the distributions of this last metric. In a significant proportion of isotropic event cases, CN shows a lower average delay than LA-EXP. However, with larger underlying event anisotropy the performance difference increases in favor of LA-EXP. In this way, the results appear to confirm previous observations and show that the average results are a reliable indication of the relative performance of each algorithm.

**Figure 8:**
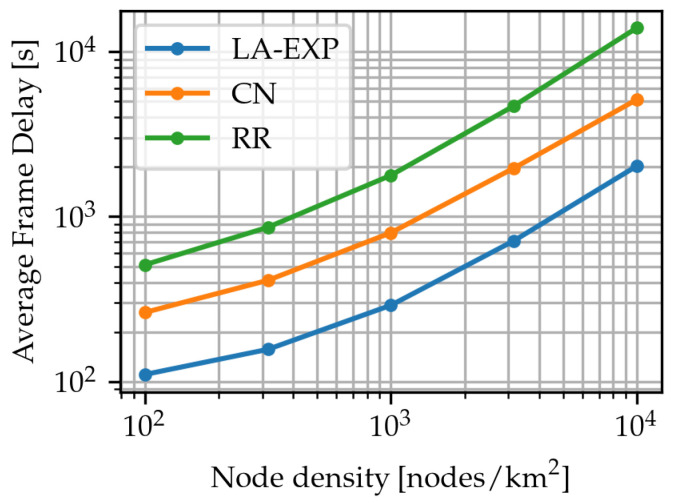
Expected average frame delay of the RR, CN, and LA-EXP algorithms for variable network node deployment density λ.

As a final question, to investigate the impact of network scale on scheduling performance, the simulation was run with all three scheduling algorithms under the thin-type event, for scheduler parameter values minimizing frame loss ratio, and for network node deployment density values λ ranging in a logarithmic scale from $10^{2}$ to $10^{4}$ nodes/km^2^. As a remark, with varying node density, the number of nodes belonging to each cluster, and thus the time required to schedule one cluster, changes proportionally with the node density. For each point, the expected average frame delay was computed from n=500 instances. The results, shown in Figure 8, indicate a slightly super-linear relationship between network node density and average frame delay. It is clear that LA-EXP maintains its advantage at larger network scales compared to CN and RR.

## 6. Discussion, Limitations, and Future Work

To overcome the shortcomings of Closest Next Scheduling, a novel spatial scheduling algorithm has been developed and is introduced in this paper. Closest Next Scheduling is to some extent adapted to a specific event-triggered traffic model, and as such, its performance varies not only with the scale of the event (i.e., the load of the event report traffic that is generated) but also with the spatial shape of the event’s area of effects, generally favoring circular shaped events. Other cases that the novel algorithm is expected to be able to successfully tackle are multiple events and events that spread over an underlying graph topology, rather than Euclidean space. The latter occasion would arise, for example, if an event affected a core node on the electric power grid as well as its dependent smaller units. This, however, requires further investigation.

Another interesting point is that the proposed algorithm’s behavior is essentially a search over the *m*-nearest neighbor graph induced by the physical placement of the network sensors, as represented in the distance matrix D. Using the learning automaton, the scheduler explores the area around the event by following links on that graph, without extending beyond the area of the event. Thus, its operation and result are independent of the geometric shape of the event’s area of effect, which also includes the case of multiple simultaneous events. The problem of finding the optimal LA-EXP *m* value is directly related to the *m*-nearest neighbor graph connectivity problem. This is because, in order for the LA-EXP learning automaton to successfully explore the graph, it is required that the nodes are linked to their neighbors in every direction. While previous works on the connectivity of *m*-nearest neighbor graphs have yielded bounds on the value of *m*, required so that a randomly generated *m*-nearest neighbor graph is connected with high probability [38], these bounds seem to require a higher *m* value for higher node counts, which is counter-intuitive as, in this case, the value of *m* to achieve a specific performance level decreases as node density increases. The study of this problem is an open question, which directly relates to the problem of analytically modeling the scheduling algorithm used in this work as well.

At a more general level, by demonstrating the feasibility and potential performance benefit of the approach, this work lays the foundation for a learning-based adaptive spatial paradigm in wireless networking, which focuses on performance optimization based on network geometry and topology. In this way, the way is paved for applications in higher-level networking layers, such as the routing layer. As an example, works on routing for wireless sensor networks (WSN)—like [39], where a fuzzy logic-based congestion classifier is used to control service rate allocation between local and transit traffic in a WSN to increase performance, or [40], where a WSN clustered routing algorithm is developed aiming to increase energy efficiency—could attain further performance benefits by deploying algorithms following the adaptive spatial paradigm, if the traffic exhibits spatial correlation behavior.

Furthermore, with the pervasive and increasing IoT network deployment across critical applications such as healthcare and infrastructure monitoring, the imperative to fortify these networks against evolving security threats becomes increasingly relevant, given the ever-growing demand for dependable and secure communication in the IoT era. Compared to classical networks, resource-constrained IoT networks pose an additional challenge, as they require a balance between robust protection and resource efficiency through lightweight security solutions. Recent works in this area include [41], which focuses on the problem of IoT network security in the space of healthcare, and [42], which focuses on the performance comparison of two cryptographic functions, thus highlighting the importance of resource efficiency. A question that arises from the present work is whether using adaptive spatial techniques increases security through energy efficiency and network reliability, or introduces security vulnerabilities, in which case they would need to be addressed.

While this work proposes an answer to the adaptive spatial scheduling problem, a few questions remain open. First, the mechanism that is used by the network server to detect the event is left unspecified. Second, in this work, the nodes are assumed to be stationary and, therefore, device positions can be recorded and transmitted to the network server. Thus, the case of mobile sensors remains open.

## 7. Conclusions

In this paper, a novel adaptive spatial scheduling algorithm, called Learning Automaton-based Exploration (LA-EXP) scheduling, targeting spatially auto-correlated event-triggered traffic on LoRaWAN networks was introduced. The algorithm uses a learning automaton to map the event area of effect based on network feedback information, and thus estimate the probability of each sensor group being ready to send event-related information. To assess the performance characteristics of the newly proposed algorithm, a simulation study was carried out, where the algorithm was evaluated on its own, but also against a previously proposed algorithm, Closest Next (CN). The study showed that LA-EXP achieves similar performance to CN in the case of isotropic events, but as the area of effect of an event becomes more anisotropic, LA-EXP takes better advantage of the particular event shape and achieves the same frame loss at a lower average frame delay. The algorithm is also more robust, as its performance depends on its parameterization only up to a certain value, after which the minimum frame loss is achieved stably.

## Figures and Tables

**Table 1:** Notation summary.

Notation	Description
$\Psi \left(x\right),\;\;x\in R^{2}$	Event area of effect function
*s*	Size factor of random event generation
*d*	Directional bias strength of random event generation
*S*	Node set
N=|S|	Number of nodes
λ	Node density of the network
$D={\left(d_{ij}\right)}_{N\times N}$	Node distance matrix
$w,\;\;w_{i},\;i\in S$	Node weight vector and node weights
$q,\;\;q_{g},\;g\in G$	Group weight vector and group weights
$u_{i}\left(t\right),\;i\in S$	Penalty factor
$\eta_{in},\;i,n\in S$	Conditional probability that node *i* has generated an event given that node *n* has generated an event
φ	Kernel function used in modelling $\eta_{in}$
*m*	Minimum updated neighbors parameter of LA-EXP
$r_{n;m}$	Distance of node *n* from its *m*-th closest neighbor
$I_{\max}$	Maximum idle time parameter of CN scheduling [17]

**Table 2:** Event types considered in the simulations.

**Event Type**	**Event Size** *s*	**Directional** **Strength** *d*	**Notes**
Isotropic	25	0	Small size isotropic event. Favours CN the most.
Thin	700	30	Elongated event. Favours CN the least.
Large	1000	10	Less anisotropic event with a larger area of effect.

## Data Availability

The raw data supporting the conclusions of this article will be made available by the authors on request.

## References

[1] Qadir Q.M., Rashid T.A., Al-Salihi N.K., Ismael B., Kist A.A., Zhang Z. (2018). Low Power Wide Area Networks: A Survey of Enabling Technologies, Applications and Interoperability Needs. IEEE Access.

[2] Laner M., Svoboda P., Nikaein N., Rupp M. Traffic Models for Machine Type Communications. Proceedings of the Tenth International Symposium on Wireless Communication Systems (ISWCS 2013).

[3] Technical Specification Group Radio Access Network (2011). Study on RAN Improvements for Machine-Type Communications.

[4] Gupta V., Devar S.K., Kumar N.H., Bagadi K.P. Modelling of IoT Traffic and Its Impact on LoRaWAN. Proceedings of the GLOBECOM 2017—2017 IEEE Global Communications Conference.

[5] Kaburaki A., Adachi K., Takyu O., Ohta M., Fujii T. (2021). Autonomous Decentralized Traffic Control Using Q-Learning in LPWAN. IEEE Access.

[6] Tsakmakis A., Valkanis A., Beletsioti G., Kantelis K., Nicopolitidis P., Papadimitriou G. (2022). An Adaptive LoRaWAN MAC Protocol for Event Detection Applications. Sensors.

[7] Asteriou V., Valkanis A., Beletsioti G., Kantelis K., Papadimitriou G., Nicopolitidis P. (2022). LoRaWAN-Based Adaptive MACs for Event Response Applications. IEEE Access.

[8] Zorbas D., Abdelfadeel K.Q., Cionca V., Pesch D., O’Flynn B. Offline Scheduling Algorithms for Time-Slotted LoRa-based Bulk Data Transmission. Proceedings of the 2019 IEEE 5th World Forum on Internet of Things (WF-IoT).

[9] Abdelfadeel K.Q., Zorbas D., Cionca V., Pesch D. (2020). FREE —Fine-Grained Scheduling for Reliable and Energy-Efficient Data Collection in LoRaWAN. IEEE Internet Things J..

[10] Haxhibeqiri J., Moerman I., Hoebeke J. (2019). Low Overhead Scheduling of LoRa Transmissions for Improved Scalability. IEEE Internet Things J..

[11] Garrido-Hidalgo C., Haxhibeqiri J., Moons B., Hoebeke J., Olivares T., Ramirez F.J., Fernández-Caballero A. (2021). LoRaWAN Scheduling: From Concept to Implementation. IEEE Internet Things J..

[12] Xu Z., Luo J., Yin Z., He T., Dong F. S-MAC: Achieving High Scalability via Adaptive Scheduling in LPWAN. Proceedings of the IEEE INFOCOM 2020—IEEE Conference on Computer Communications.

[13] Chinchilla-Romero N., Navarro-Ortiz J., Muñoz P., Ameigeiras P. (2021). Collision Avoidance Resource Allocation for LoRaWAN. Sensors.

[14] Kaburaki A., Adachi K., Takyu O., Ohta M., Fujii T. (2023). Adaptive Resource Allocation Utilizing Periodic Traffic and Clock Drift in LPWAN. IEEE Trans. Wirel. Commun..

[15] El Fehri C., Baccour N., Kammoun I. (2023). A New Schedule-Based Scheme for Uplink Communications in LoRaWAN. IEEE Open J. Commun. Soc..

[16] Shen L.H., Wu C.H., Su W.C., Feng K.T. (2021). Analysis and Implementation for Traffic-Aware Channel Assignment and Contention Scheme in LoRa-Based IoT Networks. IEEE Internet Things J..

[17] Asteriou V., Papadimitriou G., Nicopolitidis P. Adaptive MAC Protocols for IoT Edge Computing Architectures with Event-Triggered Traffic. Proceedings of the 2021 IEEE International Black Sea Conference on Communications and Networking (BlackSeaCom).

[18] Piyare R., Murphy A.L., Magno M., Benini L. (2018). On-Demand LoRa: Asynchronous TDMA for Energy Efficient and Low Latency Communication in IoT. Sensors.

[19] Frøytlog A., Haglund M.A., Cenkeramaddi L.R., Beferull-Lozano B. Design and implementation of a long-range low-power wake-up radio and customized DC-MAC protocol for LoRaWAN. Proceedings of the 2019 IEEE International Conference on Advanced Networks and Telecommunications Systems (ANTS).

[20] Slats L. A Brief History of LoRa: Three Inventors Share Their Story, at Inside Out: Semtech’s Corporate Blog. https://blog.semtech.com/a-brief-history-of-lora-three-inventors-share-their-personal-story-at-the-things-conference.

[21] Banti K., Karampelia I., Dimakis T., Boulogeorgos A.A.A., Kyriakidis T., Louta M. (2022). LoRaWAN Communication Protocols: A Comprehensive Survey under an Energy Efficiency Perspective. Telecom.

[22] Vangelista L. (2017). Frequency Shift Chirp Modulation: The LoRa Modulation. IEEE Signal Process. Lett..

[23] Croce D., Gucciardo M., Mangione S., Santaromita G., Tinnirello I. (2018). Impact of LoRa Imperfect Orthogonality: Analysis of Link-Level Performance. IEEE Commun. Lett..

[24] Technical Specification Group GSM/EDGE Radio Access Network (2015). Cellular System Support for Ultra-Low Complexity and Low Throughput Internet of Things (CIoT).

[25] Corrales Madueño G., Stefanović C., Popovski P. (2016). Reliable and Efficient Access for Alarm-Initiated and Regular M2M Traffic in IEEE 802.11ah Systems. IEEE Internet Things J..

[26] Fox J.M., Whitesides G.M. (2015). Warning signals for eruptive events in spreading fires. Proc. Natl. Acad. Sci. USA.

[27] El abbassi M.A., Jilbab A., Bourouhou A. (2020). Efficient Forest Fire Detection System Based on Data Fusion Applied in Wireless Sensor Networks. Int. J. Electr. Eng. Inform..

[28] Mousavi A., Duckham M., Kotagiri R., Rajabifard A. Spatio-temporal event detection using probabilistic graphical models (PGMs). Proceedings of the 2013 IEEE Symposium on Computational Intelligence and Data Mining (CIDM).

[29] Bridson R. Fast Poisson disk sampling in arbitrary dimensions. Proceedings of the ACM SIGGRAPH 2007 Sketches. Association for Computing Machinery (SIGGRAPH’07).

[30] Saelens M., Hoebeke J., Shahid A., Poorter E.D. (2019). Impact of EU duty cycle and transmission power limitations for sub-GHz LPWAN SRDs: An overview and future challenges. J. Wirel. Commun. Netw..

[31] LoRa Alliance (2020). LoRaWAN® L2 1.0.4 Specification.

[32] Crippen G.M. (1978). Note rapid calculation of coordinates from distance matrices. J. Comput. Phys..

[33] Dokmanic I., Parhizkar R., Ranieri J., Vetterli M. (2015). Euclidean Distance Matrices: Essential theory, algorithms, and applications. IEEE Signal Process. Mag..

[34] Papadimitriou G., Obaidat M., Pomportsis A. (2002). On the use of learning automata in the control of broadcast networks: A methodology. IEEE Trans. Syst. Man, Cybern. Part B (Cybern.).

[35] Beletsioti G.A., Papadimitriou G.I., Nicopolitidis P., Varvarigos E., Mavridopoulos S. (2020). A Learning-Automata-Based Congestion-Aware Scheme for Energy-Efficient Elastic Optical Networks. IEEE Access.

[36] Van den Abeele F., Haxhibeqiri J., Moerman I., Hoebeke J. (2017). Scalability Analysis of Large-Scale LoRaWAN Networks in NS-3. IEEE Internet Things J..

[37] Lim J.T., Han Y. (2018). Spreading Factor Allocation for Massive Connectivity in LoRa Systems. IEEE Commun. Lett..

[38] Balister P., Bollobás B., Sarkar A., Walters M. (2005). Connectivity of Random k-Nearest-Neighbour Graphs. Adv. Appl. Probab..

[39] Hatamian M., Almasi Bardmil M., Asadboland M., Hatamian M., Barati H. (2016). Congestion-Aware Routing and Fuzzy-based Rate Controller for Wireless Sensor Networks. Radioengineering.

[40] Dehkordi E.G., Barati H. (2023). Cluster based routing method using mobile sinks in wireless sensor network. Int. J. Electron..

[41] Sundas A., Badotra S., Bharany S., Almogren A., Tag-ElDin E.M., Rehman A.U. (2022). HealthGuard: An Intelligent Healthcare System Security Framework Based on Machine Learning. Sustainability.

[42] Adeniyi E.A., Falola P.B., Maashi M.S., Aljebreen M., Bharany S. (2022). Secure Sensitive Data Sharing Using RSA and ElGamal Cryptographic Algorithms with Hash Functions. Information.

